# Trends in stroke incidence rates and stroke risk factors in Rotterdam, the Netherlands from 1990 to 2008

**DOI:** 10.1007/s10654-012-9673-y

**Published:** 2012-03-17

**Authors:** Renske G. Wieberdink, M. Arfan Ikram, Albert Hofman, Peter J. Koudstaal, Monique M. B. Breteler

**Affiliations:** 1 Department of Epidemiology, Erasmus MC University Medical Center, P.O. Box 2040, 3000 CA Rotterdam, The Netherlands; 2 Department of Neurology, Erasmus MC University Medical Center, Rotterdam, The Netherlands; 3 Department of Radiology, Erasmus MC University Medical Center, Rotterdam, The Netherlands; 4 DZNE, German Center for Neurodegenerative Diseases, Bonn, Germany

**Keywords:** Stroke, Epidemiology, Incidence, Cohort study, Risk factors, Trends

## Abstract

Stroke incidence rates have decreased in developed countries over the past 40 years, but trends vary across populations. We investigated whether age-and-sex-specific stroke incidence rates and associated risk factors as well as preventive medication use have changed in Rotterdam in the Netherlands during the last two decades. The study was part of the Rotterdam Study, a large population-based cohort study among elderly people. Participants were 10,994 men and women aged 55–94 years who were stroke-free at baseline. Trends were calculated by comparing the 1990 subcohort (*n* = 7516; baseline 1990–1993) with the 2000 subcohort (*n* = 2883; baseline 2000–2001). Poisson regression was used to calculate incidence rates and incidence rate ratios in age-and-sex-specific strata. We further compared the prevalence of stroke risk factors and preventive medication use in the two subcohorts. In the 1990 subcohort 467 strokes occurred during 45,428 person years; in the 2000 subcohort 115 strokes occurred in 18,356 person years. Comparing the subcohorts, incidence rates decreased by 34% in men, but remained unchanged in women. Blood pressure levels increased between 1990 and 2000, whereas the proportion of current cigarette smokers decreased in men, but not in women. There was a strong increase in medication use for treatment of stroke risk factors across all age categories in both sexes. Our findings suggest that in Rotterdam between 1990 and 2008 stroke incidence rates have decreased in men but not in women.

## Introduction

The worldwide burden of stroke is predicted to increase due to increased life expectancy and population aging [1]. On the other hand, in several high-income countries, stroke mortality trends were predicted to decrease in the decades ahead [2]. Furthermore, age-specific stroke incidence rates have decreased in high-income countries during the past 40 years [3]. However, there are substantial geographic differences in stroke incidence rates and trends over time [4]. Currently it is not clear if stroke incidence rates are changing in the Netherlands.

Stroke is increasingly recognized as a disease that can be prevented [5]. Knowledge of modifiable risk factors and the availability of primary preventive medication has resulted in the development of evidence-based guidelines for the treatment of high-risk individuals [6]. Furthermore, public health campaigns were initiated to improve awareness of risk factors and to promote lifestyle changes on a population level. Whether these interventions resulted in a favorable risk factor profile of the general population and, in parallel, in a reduction in stroke events is unknown and requires investigation. Additionally, studies suggest that women may receive suboptimal treatment of cardiovascular risk factors because of lower perception of risk by treating physicians [7]. Therefore sex-specific changes in risk factor profiles and stroke incidence rates are of particular interest.

The aim of the present study was to describe temporal trends in stroke incidence rates in the Netherlands during the past 20 years, and to investigate changes in preventive medication use and the prevalence and severity of stroke risk factors. We particularly aimed to compare patterns between men and women. All analyses were based on data from the Rotterdam Study, a large prospective population-based cohort study among elderly people living in the city of Rotterdam in the Netherlands.

## Methods

### Study population

The Rotterdam Study is an ongoing prospective population-based cohort study that focuses on causes and consequences of chronic and disabling diseases in the elderly [8]. The cohort started in 1990 and included 7,983 participants aged ≥55 years living in Ommoord, a district of the city of Rotterdam in the Netherlands (participation rate 78%). In 2000, the cohort was expanded with 3,011 people who had reached the age of 55 or had moved into the district since the start of the study (response rate 67%). The study was approved by the Medical Ethics Committee of the Erasmus MC University Medical Center and all participants gave written informed consent to participate in the study.

The present study included participants of the 1990 subcohort and the 2000 subcohort who were aged 55–94 at baseline (*n* = 10944). Participants who had had a stroke before baseline (*n* = 361) and participants who had not signed consent for the collection of follow-up data from general practitioners (*n* = 184) were excluded. This resulted in 10,399 participants in the population for analysis (1990 subcohort, *n* = 7,516; 2000 subcohort, *n* = 2,883).

### Stroke risk factors and preventive medication use

Trained research physicians visited all participants at home for filling in standardized questionnaires about their health status, medical history and current medication use. Data on female hormone use, including both oral contraceptive use and hormone replacement therapy for menopausal complaints, were collected at baseline and classified as current use or ever use. Subsequently, participants visited the research center twice for physical examinations and blood sampling. Cigarette smoking behavior was classified as current, past or never. Blood pressure was measured twice at the right brachial artery with a random-zero sphygmomanometer after 5 min of rest while the subject was in a sitting position. Hypertension grade I was defined as a diastolic blood pressure of 90–99 mm Hg or a systolic blood pressure of 140–159 mm Hg. Hypertension grade II was defined as a diastolic or systolic blood pressure of ≥100/160 mm Hg, and/or use of blood pressure-lowering medication [9]. Total serum cholesterol, high-density lipoprotein cholesterol and glucose levels were measured using an automated enzymatic procedure. Non-fasting blood samples were obtained from the 1990 subcohort, fasting blood samples from the 2000 subcohort. Diabetes mellitus was defined as a fasting glucose level ≥7.0 mmol/L, a non-fasting or post-load serum glucose level ≥11.1 mmol/L and/or the use of blood glucose-lowering drugs. Body mass index was calculated as weight (in kilograms) divided by the square of height (in meters). Atrial fibrillation was diagnosed when observed on ECG during the visit to the research center or when reported in medical records. History of myocardial infarction was considered positive when self-reported during the interview, observed on ECG during the center visit, and/or confirmed in medical records.

### Assessment of stroke

Stroke was defined according to WHO criteria as a syndrome of rapidly developing clinical signs of focal (or global) disturbance of cerebral function, with symptoms lasting 24 h or longer or leading to death, with no apparent cause other than of vascular origin [10]. History of stroke at baseline was assessed during the baseline interview and verified by reviewing medical records. After enrollment, participants were continuously monitored for incident stroke through automated linkage of the study database with files from general practitioners. Nursing home physicians’ files and files from general practitioners of participants who moved out of the district were checked on a regular basis as well. Additional information was obtained from hospital records. Potential strokes were reviewed by research physicians, and verified by an experienced stroke neurologist (P.J.K.). Strokes were further classified as cerebral infarction or intracerebral hemorrhage based on neuroimaging reports. If neuroimaging was lacking, a stroke was classified as unspecified. This classification corresponded with ICD-10 codes I61, I63 and I64. Transient ischemic attacks or subarachnoid hemorrhages were not included. Participants could contribute person years to the follow-up for a maximum of 7 years, that is, from baseline until a first-ever stroke occurred, or until death, or until they reached the age of 95, or, if lost to follow-up, until their last health status update when they were known to be free of stroke, whichever came first, or until they completed 7 years of follow-up. The follow-up of the 1990 subcohort was complete for 99.8% of potential person years, the follow-up of the 2000 subcohort for 97.8% of potential person years.

### Statistical analysis

We compared the prevalence of risk factors and preventive medication use between the 1990 and the 2000 subcohorts, in strata of men and women, and in 10 year age categories. ANCOVA was used to calculate age-adjusted *P* values for the differences between cohorts. We used Poisson regression to calculate age-adjusted incidence rates and rate ratios in 10 year age strata and in the total population aged 55–94. The 1990 subcohort was used as the reference cohort. During the follow-up period, participants could move to a higher age category, i.e., we used a dynamic cohort population. Therefore, participants could contribute person years to subsequent age categories and could be included in the “number at risk” in more than one age category. Stroke events were counted in the age category the participant belonged to at the moment the stroke occurred. We further assessed whether overall stroke incidence rate ratios differed between men and women by including a multiplicative interaction term (sex * subcohort) in the regression model and we calculated incidence rates and rate ratios in sex-specific strata.

## Results

The prevalence of stroke risk factors and preventive medication use in the 1990 and the 2000 subcohorts are presented in Table 1 for men and in Table 2 for women. The prevalence of grade II hypertension increased with age and was higher in women than in men within both cohorts. Comparing the 1990 with the 2000 subcohort, we observed an enormous increase in grade II hypertension over time, due to an increase of both systolic and diastolic blood pressure. Blood pressure-lowering medication use had not increased over time, except for men aged 85–94. The prevalence of diabetes mellitus and blood glucose-lowering medication use had increased only in the youngest age groups (men and women aged 55–64) but remained stable in the other strata. Serum HDL-cholesterol levels were higher in women than in men in all age categories. HDL-cholesterol levels did not vary with age and did not differ over time. The 2000 subcohort more often used lipid-lowering medication than the 1990 subcohort. However, in both subcohorts, lipid-lowering medication use was more common in the younger than in the older age groups and more common in men than in women. Body mass index increased over time and was higher in women than in men. The proportion of current cigarette smokers decreased in men, but remained more or less stable in women. In the 1990 subcohort, cigarette smoking was much more frequent in men than in women, but in the 2000 subcohort the proportion of cigarette smokers among men and women was almost the same. Men more often had a history of myocardial infarction than women. From 1990 to 2000, the prevalence of myocardial infarction had decreased in men aged 55–64, but was unchanged in other age and sex strata. The presence of atrial fibrillation increased with age but did not change over time. Antithrombotic medication use increased with age and increased enormously over time both in men and women. We further investigated the use of sex hormones in women. Though current female hormone use was uncommon in both subcohorts, the proportion of women that had ever taken female hormones, either oral contraceptives or hormone replacement therapy, had increased enormously over time.

**Table 1:** Prevalence of stroke risk factors and preventive medication use in men

Age category Subcohort No.	55–64			65–74			75–84			85–94		
	1990	2000	*P*	1990	2000	*P*	1990	2000	*P*	1990	2000	*P*
	*N* = 1,144	*N* = 822		*N* = 1,125	*N* = 277		*N* = 557	*N* = 130		*N* = 122	*N* = 22	
Age, years	60.4 (2.8)	59.8 (2.5)	<0.001	69.5 (2.9)	69.5 (2.8)	0.99	79.1 (2.8)	79.0 (3.0)	0.68	88.3 (2.4)	87.7 (2.5)	0.35
SBP, mm Hg	135 (21)	141 (20)	<0.001	140 (21)	148 (23)	<0.001	142 (23)	153 (22)	<0.001	144 (26)	145 (24)	0.96
DBP, mm Hg	76 (11)	82 (11)	<0.001	75 (12)	80 (11)	<0.001	72 (12)	77 (12)	<0.001	71 (13)	73 (11)	0.78
*Hypertension*, *%*												
No	54.4	42.9	<0.001	42.7	31.9	<0.001	40.8	22.6	<0.001	41.8	21.1	0.101
Grade I	20.1	26.2	<0.001	23.9	26.5	0.37	24.9	26.1	0.79	29.6	31.6	0.89
Grade II	25.5	30.9	<0.001	33.4	41.5	0.01	34.3	51.3	<0.001	28.6	47.4	0.12
Diabetes mellitus, %	7.2	10.7	0.01	11.4	13.8	0.28	13.9	17.1	0.36	18.8	14.3	0.71
HDL, mmol/L	1.2 (0.3)	1.2 (0.3)	0.70	1.2 (0.3)	1.2 (0.3)	0.30	1.2 (0.3)	1.3 (0.3)	0.09	1.3 (0.3)	1.3 (0.4)	0.32
*Cigarette smoking, %*												
Current	29.0	23.7	0.01	23.4	16.3	0.01	20.8	10.1	0.01	20.2	4.5	0.08
Past	55.8	55.1	0.96	63.5	70.7	0.03	56.9	72.1	<0.001	39.5	81.8	<0.001
Never	15.2	21.2	<0.001	13.0	13.0	0.99	22.3	17.8	0.27	40.4	13.6	0.02
Myocardial infarction, %	8.0	3.3	<0.001	13.1	10.8	0.30	13.5	9.2	0.19	8.6	13.6	0.41
Atrial fibrillation, %	1.6	1.1	0.40	6.1	5.4	0.67	12.9	8.5	0.17	16.9	22.7	0.50
Body mass index, kg/m^2^	25.8 (2.9)	27.2 (3.4)	<0.001	25.7 (2.9)	26.7 (3.2)	<0.001	25.2 (3.2)	25.9 (3.0)	0.04	24.3 (3.6)	25.3 (2.9)	0.26
*Preventive medication use*, *%*												
Blood pressure- lowering	22.2	22.5	0.64	30.4	30.7	0.93	36.3	36.9	0.89	38.8	54.5	0.19
Lipid-lowering	3.4	12.7	<0.001	2.7	16.6	<0.001	0.5	9.2	<0.001	0.8	4.5	0.20
Antithrombotic	3.9	12.2	<0.001	9.6	30.0	<0.001	8.1	32.3	<0.001	9.9	40.9	<0.001
Glucose-lowering	2.5	4.0	0.04	5.7	7.2	0.34	5.2	10.0	0.04	4.1	9.1	0.27

**Table 2:** Prevalence of stroke risk factors and preventive medication use in women

Age category Subcohort No.	55–64			65–74			75–84			85–94		
	1990	2000	*P*	1990	2000	*P*	1990	2000	*P*	1990	2000	*P*
	*N* = 1,537	*N* = 1,059		*N* = 1,500	*N* = 282		*N* = 1,055	*N* = 229		*N* = 476	*N* = 62	
Age, years	60.2 (2.8)	60.0 (2.5)	0.01	70.0 (2.8)	69.8 (3.0)	0.38	79.5 (2.9)	79.2 (2.7)	0.10	88.5 (2.6)	87.6 (2.0)	0.01
SBP, mm Hg	131 (21)	137 (19)	<0.001	142 (21)	149 (23)	<0.001	148 (23)	159 (23)	<0.001	148 (24)	163 (24)	<0.001
DBP, mm Hg	74 (11)	78 (10	<0.001	73 (11)	78 (11)	<0.001	73 (12)	78 (11)	<0.001	71 (14)	78 (10)	0.01
*Hypertension*, *%*												
No	58.0	47.7	<0.001	36.7	28.9	0.01	26.6	13.2	<0.001	24.9	11.4	0.07
Grade I	15.5	24.2	<0.001	22.9	23.0	0.97	23.0	23.4	0.98	23.5	15.9	0.22
Grade II	26.4	28.1	0.20	40.3	48.0	0.02	50.5	63.4	<0.001	51.7	72.7	0.01
Diabetes mellitus, %	4.8	7.7	<0.001	10.4	11.8	0.50	16.5	14.0	0.42	17.9	13.1	0.28
HDL, mmol/L	1.5 (0.4)	1.5 (0.4)	<0.001	1.4 (0.4)	1.4 (0.4)	0.39	1.4 (0.4)	1.4 (0.4)	0.12	1.4 (0.4)	1.5 (0.3)	0.03
*Cigarette smoking*, *%*												
Current	25.8	24.5	0.37	18.3	17.1	0.56	10.4	9.2	0.49	3.3	6.6	0.32
Past	32.2	37.5	<0.001	28.6	43.1	<0.001	22.3	38.4	<0.001	15.6	32.8	<0.001
Never	42.0	38.0	0.04	53.0	39.9	<0.001	67.3	52.4	<0.001	81.1	60.7	<0.001
Myocardial infarction, %	1.2	1.1	0.97	3.6	2.8	0.56	5.9	3.1	0.11	5.0	9.7	0.12
Atrial fibrillation, %	0.8	0.9	0.61	4.0	2.5	0.22	8.5	10.5	0.30	17.1	6.5	0.03
Body mass index, kg/m^2^	26.3 (0.4)	27.4 (4.6)	<0.001	27.1 (4.1)	27.6 (4.6)	0.04	26.9 (4.2)	27.8 (4.4)	0.01	26.4 (4.2)	26.5 (4.1)	0.88
*Preventive medication use*, *%*												
Blood pressure-lowering	21.7	22.5	0.46	35.2	36.5	0.61	46.6	47.2	0.85	53.8	41.9	0.08
Lipid-lowering	2.6	10.5	<0.001	3.1	13.8	<0.001	0.9	12.7	<0.001	0.4	1.6	0.29
Antithrombotic	1.3	5.3	<0.001	3.7	14.2	<0.001	5.4	26.2	<0.001	5.5	40.3	<0.001
Glucose-lowering	1.7	2.9	0.03	4.9	6.0	0.43	8.1	7.4	0.76	6.5	11.3	0.21
*Female hormone use*, *%*												
Current	4.9	10.7	<0.001	1.0	2.8	0.01	0.5	1.7	0.04	0.0	1.6	0.01
Ever	57.2	85.3	<0.001	30.4	67.0	<0.001	8.5	35.0	<0.001	3.1	16.0	<0.001

Values are means (SD) or percentages. *P* values are adjusted for age. *SBP* systolic blood pressure, *DBP* diastolic blood pressure, *HDL* high-density lipoprotein cholesterol level, *BMI* body mass index, *WHR* waist-to-hip ratio

**Fig. 1:**
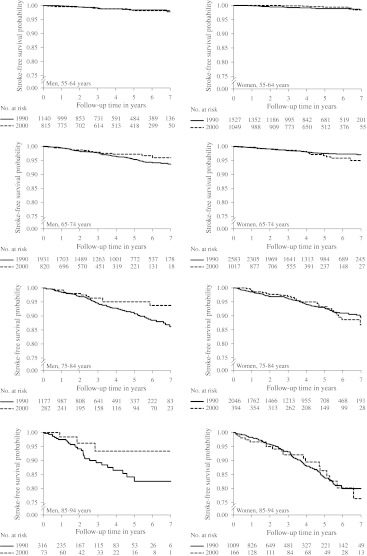
Age-and-sex-specific Kaplan–Meier curves for the stroke-free survival of the 1990 versus the 2000 subcohort

Fig. 1 shows age-and-sex-specific Kaplan–Meier curves for the stroke-free survival in the 1990 versus the 2000 subcohort. Age-adjusted stroke incidence rates and incidence rate ratios are shown in Table 3. In the total population including both men and women, we observed no change in age-adjusted stroke incidence rates. However, after stratification for sex, we found that the change in incidence rate ratios was different in men and women (*P*_interaction_ = 0.04). In men, stroke incidence rates were unchanged in the 55–64 year age category, but decreased in the 65–74, 75–84 and 85–94 year age categories. In all men (55–94), the overall stroke incidence rate had decreased with 34%. In women, age-specific stroke incidence rates showed a variable pattern. Incidence rates decreased in women aged 55–64, increased in women aged 65–74, and were stable in women aged 75–84 and 85–94. In all women (55–94), stroke incidence rates had not changed over time.

**Table 3:** Stroke incidence rates and rate ratios

	1990 Subcohort				2000 Subcohort				
	At risk N*	Events *n*	Person years	Incidence rate/1,000 person years	At risk N*	Events *n*	Person years	Incidence rate/1,000 person years	Incidence rate ratio (95% CI)
*Total population*									
55–64	2,667	28	11289.9	2.65	1,864	19	8689.9	2.33	0.88 (0.49–1.58)
65–74	4,514	120	18261.6	5.77	1,837	39	6263.1	6.43	1.11 (0.77–1.61)
75–84	3,223	196	11845.6	16.44	676	36	2628.7	13.58	0.83 (0.58–1.18)
85–94	1,325	123	4031.1	27.71	2,39	21	774.8	25.85	0.93 (0.59–1.48)
All ages	7,516	467	45428.2	7.70	2,883	115	18356.5	6.85	0.89 (0.72–1.10)
*Men*									
55–64	1,140	14	4755.1	3.32	815	11	3826.5	3.30	1.00 (0.45–2.20)
65–74	1,931	72	7883.0	8.47	820	18	2824.2	6.46	0.76 (0.45–1.29)
75–84	1,177	79	4133.5	18.90	282	11	1024.6	10.62	0.56 (0.30–1.06)
85–94	316	28	845.0	28.05	73	3	217.0	12.95	0.46 (0.14–1.53)
All ages	2,948	193	17616.6	9.01	1,251	43	7892.2	5.95	0.66 (0.47–0.92)
*Women*									
55–64	1,527	14	6534.8	2.05	1,049	8	4863.4	1.58	0.77 (0.32–1.84)
65–74	2,583	48	10378.6	3.70	1,017	21	3439.0	6.34	1.71 (1.01–2.90)
75–84	2,046	117	7712.1	15.10	394	25	1604.1	15.47	1.02 (0.66–1.58)
85–94	1,009	95	3186.1	27.61	166	18	557.8	30.89	1.12 (0.68–1.85)
All ages	4,568	274	27811.6	6.79	1,632	72	10464.3	7.38	1.09 (0.83–1.41)

Incidence rates and incidence rate ratios are adjusted for age

* Persons could contribute person years to more than one age category

## Discussion

We investigated trends in stroke incidence in Rotterdam, the Netherlands from 1990 to 2008 by comparing two subcohorts of the large population-based Rotterdam Study. We also investigated changes in stroke risk factors and preventive medication use. We found that stroke incidence rates decreased by 34% in men, but remained unchanged in women. In men, but not in women, the proportion of current cigarette smokers had decreased. However, both in men and women, blood pressure levels had increased, whereas blood pressure lowering medication use was unchanged. Body mass index also increased in men and women. History of female hormone use, either oral contraceptives or hormone replacement therapy, had increased over time. The use of lipid-lowering medication and antithrombotic medication had increased enormously both in men and women.

This study has several strengths, including the prospective and population-based design and the large number of participants. Furthermore, we compared two subcohorts with the same study design and inclusion criteria at two different time-periods (1990–1998 versus 2000–2008). Thorough stroke monitoring procedures and the nearly complete follow-up allowed us to include virtually all stroke events, even in participants who had not been referred to a neurologist or admitted to a hospital, for example people living in nursing homes or participants who had a fatal stroke. An advantage of this approach was that the trends we observed have not been influenced by changes in referral or admission patterns. However, a disadvantage was that neuroimaging had not been performed in all stroke patients. Because the 2000 subcohort had more widely access to brain CT or MRI than the 1990 subcohort, a larger proportion of events in the 2000 subcohort could be subclassified as ischemic or hemorrhagic and a smaller proportion remained unspecified as compared to the 1990 subcohort. As a consequence, we could not compare incidence rates of ischemic and hemorrhagic stroke subtypes.

Another potential limitation of our study is the external validity of the results obtained. The Rotterdam Study was performed in the well-defined Ommoord district of Rotterdam because the population of this suburb, composed mainly of white, middle class subjects, was considered to be representative for the average Dutch population. The participation rate of eligible persons was high (78% for the 1990 subcohort and 67% for the 2000 subcohort), minimizing the likelihood of selection bias. Therefore, we are confident that the trends we observed over time can be generalized to the Rotterdam population and even the Dutch population at large. However, we do not know whether our results also hold for populations with a different ethnic or socio-economic composition. We further should note the fact that our study participants were informed about their cardiovascular risk factors at baseline. Because this may have had a positive impact on prevention and treatment, it is possible that incidence rates obtained from our study cohort may be somewhat lower than actual incidence rates in the general population.

Many previous studies have reported stroke incidence rates and temporal trends. Incidence rates are, however, often hard to compare across studies, because of differences in study design and case ascertainment methods. Trends should be comparable if within-study case ascertainment methods have not changed over time. When we compare our findings to results of other studies covering the same time-period, most studies reported either declining [11–17] or stable stroke incidence rates [18–20]. None of the studies, except one, reported divergent trends for men and women [11]. Associated changes in risk factors were usually favorable, contradictory to the increase in blood pressure levels we observed [14, 16]. However, the ARCOS study from New Zealand also reported a decline in men but not in women, despite an increase in blood pressure levels, diabetes and body mass index, but a reduction in smoking. These finding were very similar to the results of our study, although they did not report on medication use for the treatment of stroke risk factors [11].

Previous studies have shown that guidelines for the prevention of cardiovascular disease are less strictly applied to women than men, mainly because the perceived risk is lower than the calculated risk [7]. Our findings suggest that this may also be the case in our population. Another factor that may have prohibited stroke rates to decline in women is that history of female hormone use increased over time. Because previous studies have shown that hormone replacement therapy is associated with a higher risk of stroke in post-menopausal women [21], this increase may have attributed to the unfavorable risk profile in women. Furthermore, although blood pressure levels increased both in men and women, grade II hypertension was much more frequent in women than men. This also may have influenced stroke incidence rates in women. Nevertheless, the decline in stroke incidence rates in men seems somewhat paradoxical to the increase in risk factors we observed, especially the increase in blood pressure levels. Based on the increase in blood pressure levels alone, one would have expected an increase in stroke incidence rates. However, a plausible explanation is that all participants in whom hypertension was diagnosed during the baseline visit were advised to visit their general practitioner for blood pressure surveillance and treatment if indicated. Therefore, blood pressure-lowering drugs will have been described to a substantial number of participants after the baseline examinations, resulting in lower population blood pressure levels during follow-up. Because thresholds for initiating blood pressure treatment decreased in the past 20 years, the beneficial effect of treatment will have been greater to the 2000 subcohort [9]. However, we also observed favorable trends, particularly the reduction in male cigarette smokers, which might have had a beneficial effect on stroke risk. The more widespread prescription of preventive medication such as lipid-lowering and antithrombotic medication may have contributed to the decreased stroke incidence as well.

In conclusion, our study suggests that in Rotterdam in the Netherlands between 1990 and 2008, stroke incidence rates have decreased in men but not in women. The findings support the notion that preventive strategies have been implemented quite successfully, at least in men, but also stress the importance of recognition and treatment of stroke risk factors in women.
